# Retraction Note: Optimal scheduling of integrated energy system based on improved grey wolf optimization algorithm

**DOI:** 10.1038/s41598-023-34197-6

**Published:** 2023-05-02

**Authors:** Jun Du, Zongnan Zhang, Menghan Li, Jing Guo, Kongge Zhu

**Affiliations:** 1 School of Energy and Power, Jiangsu University of Science and Technology, Zhenjiang, 212100 Jiangsu China; 2 State Grid Jiangsu Electric Power Co., Ltd, Lianyungang Power Supply Branch, No. 1, Xingfu Road, Haizhou District, Lianyungang CityJiangsu Province, 222000 China

Retraction of: *Scientific Reports*, 10.1038/s41598-022-10958-7, published online 02 May 2022

The Authors have retracted this Article.

Following the publication of the Article, the authors have identified a number of errors which invalidate the results reports in this paper. First, in “Case studies”, subheading “Basic data”, for the simulation the output data shown in Figure 2 were used as input. Since these are not real data, the results of the simulation are incorrect. Second, in “Methods: solving an integrated demand response IES optimization dispatch model for electricity and heat”, in the description of the application of the IGWO to the IES scheduling optimisation, in Step 4 the Authors incorrectly used the FCM clustering algorithm, which is not an appropriate solution. This makes the whole process incorrect. Finally, in “Integrated demand response model for electric and thermal multiple loads” the Authors did not consider the real-time capacity variation which makes the mathematical model incorrect.

All Authors agree with the retraction and its wording.

